# Digitally Supported Dietary Protein Counseling Changes Dietary Protein Intake, Sources, and Distribution in Community-Dwelling Older Adults

**DOI:** 10.3390/nu13020502

**Published:** 2021-02-03

**Authors:** Jantine van den Helder, Sjors Verlaan, Michael Tieland, Jorinde Scholten, Sumit Mehra, Bart Visser, Ben J. A. Kröse, Raoul H. H. Engelbert, Peter J. M. Weijs

**Affiliations:** 1 Center of Expertise Urban Vitality, Amsterdam University of Applied Sciences, 1067 SM Amsterdam, The Netherlands; m.tieland@hva.nl (M.T.); jorindescholten@gmail.com (J.S.); s.mehra@hva.nl (S.M.); b.visser2@hva.nl (B.V.); b.j.a.krose@hva.nl (B.J.A.K.); r.h.h.engelbert@hva.nl (R.H.H.E.); 2 FrieslandCampina, 3818 LE Amersfoort, The Netherlands; sjors.verlaan@frieslandcampina.com or; 3 Department of Rehabilitation Medicine, Amsterdam University Medical Centers, Vrije Universiteit, 1081 HV Amsterdam, The Netherlands; 4 CREATE-IT Applied Research, Amsterdam University of Applied Sciences, 1091 GC Amsterdam, The Netherlands; 5 Informatics Institute, University of Amsterdam, 1090 GH Amsterdam, The Netherlands; 6 Department of Rehabilitation, Amsterdam University Medical Centers, University of Amsterdam, 1105 AZ Amsterdam, The Netherlands; 7 Department of Nutrition and Dietetics, Amsterdam University Medical Centers, VU University, 1081 HV Amsterdam, The Netherlands

**Keywords:** ageing, counselling, e-health, nutrition, protein, sarcopenia

## Abstract

Digitally supported dietary counselling may be helpful in increasing the protein intake in combined exercise and nutritional interventions in community-dwelling older adults. To study the effect of this approach, 212 older adults (72.2 ± 6.3 years) were randomised in three groups: control, exercise, or exercise plus dietary counselling. The dietary counselling during the 6-month intervention was a blended approach of face-to-face contacts and videoconferencing, and it was discontinued for a 6-month follow-up. Dietary protein intake, sources, product groups, resulting amino acid intake, and intake per eating occasion were assessed by a 3-day dietary record. The dietary counselling group was able to increase the protein intake by 32% at 6 months, and the intake remained 16% increased at 12 months. Protein intake mainly consisted of animal protein sources: dairy products, followed by fish and meat. This resulted in significantly more intake of essential amino acids, including leucine. The protein intake was distributed evenly over the day, resulting in more meals that reached the protein and leucine targets. Digitally supported dietary counselling was effective in increasing protein intake both per meal and per day in a lifestyle intervention in community-dwelling older adults. This was predominantly achieved by consuming more animal protein sources, particularly dairy products, and especially during breakfast and lunch.

## 1. Introduction

As the population is ageing worldwide, the preservation of mobility, vitality, and independent living are of utmost importance for older adults. Effective exercise and nutritional interventions are recommended to support healthy and active ageing [1]. The combination of exercise training with optimal dietary protein intake has been intensively studied in randomised controlled trials [2]. These trials in older adults showed that exercise and dietary protein stimulated muscle mass gains and the effects on physical function are promising [3,4]. Although these studies show relevant benefits of protein supplementation, older adults need practical support on how to implement the recommendations of adequate protein intake into their daily diet.

There is sufficient evidence available that dietary counselling changes eating behavior [5] and may therefore be an interesting strategy to increase protein intake. However, only a small number of controlled studies are available regarding protein intake counselling [6,7,8,9], and the application of e-health and tele-health to support nutritional interventions in community-dwelling older adults [10].

The targets for protein intake for older adults range between 1.0–1.2 g/kg body weight (BW)/day for healthy and then 1.2–1.5 g/kg BW/day for older adults with acute or chronic disease [11,12]. These are challenging amounts of protein intake for many older adults. In addition to the daily protein intake, experts recommend that each meal should contain approximately 25–30 g high-quality protein or 0.4 g/kg BW/day, containing about 2.5–2.8 g of leucine, and preferably evenly distributed over the day, in order to stimulate muscle protein synthesis maximally [11,12].

The quality of the protein is an important determinant as well, especially for older adults with a reduced appetite [13]. Protein quality specifically refers to the capacity of dietary protein to deliver essential amino acids relative to their RDA and includes amino acid composition and digestibility of the protein source (as characterised by Protein Digestibility Corrected Amino Acid Score—PDCAAS— and Digestible Indispensable Amino Acid Score—DIAAS) [14]. Proteins in animal-based food products are generally acknowledged as high-quality proteins because of the high level of essential amino acids and digestibility, which are often provided in a favorable food matrix [15]. However, there is very little knowledge about which food sources and types of food products older adults consume in attempts to increase their protein intake.

Therefore, digitally supported dietary counselling as part of a combined exercise and nutritional intervention may help increase the dietary protein intake in community-dwelling older adults and may provide more insights in different aspects of their protein eating behavior. In this study, we aimed to show an increase in the protein intake by comparing the exercise plus dietary counselling group with the exercise and control groups and then examining how the increase in protein intake was established. Moreover, we were specifically interested in protein sources, product groups, resulting amino acid intake, and intake per eating occasion after the 6-month combined exercise and nutritional intervention and after the 6-month follow-up.

## 2. Materials and Methods

### 2.1. Design

The VITAMIN study was a randomised controlled trial and was conducted from March 2016 until July 2018 (Clinical Trials Registration: https://www.trialregister.nl (NTR) NL5472). Participants with a regular weekly exercise program were cluster randomised in one of the following research groups: (1) CON: Control, no additional intervention, regular lifestyle; (2) HBex: additional blended home-based exercise training; (3) HBex-Pro: additional blended home-based exercise training with dietary protein counselling.

The total study duration was 12 months, including a six-month intervention phase and a six-month follow-up. Hence, resulted in a baseline visit, effect visit (6 m) and follow-up visit (12 m). More details were described in the study protocol [16].

### 2.2. Population

The study population comprised community-dwelling older adults with a regular weekly exercise program and living in the metropolitan region of Amsterdam. Participants were eligible if they met the following criteria: (1) 55 years of age or older; (2) willingness of the general practitioner to be notified on study participation; (3) willingness to comply with the protocol in the opinion of the study physicians; (4) ability to understand the Dutch language; (5) absence of current alcohol or drug abuse in the opinion of the investigator; (6) absence of cognitive impairment, indicated by a score of ≤15 on the Mini-Mental State Examination (MMSE); and (7) absence of knee or hip surgery in the last six months [16]. Following criteria, (3) participants were excluded with e.g., cancer or degenerative neurocognitive disorders.

### 2.3. Experimental Interventions

1. Blended intervention for additional home-based exercise

The exercise intervention consisted of a blended home-based exercise program, in which we included an application on a tablet PC (e-health) and personalised coaching. More information is available in the design studies and effect study [17,18,19].

2. Dietary protein counselling incorporating e-health/tele-health

The dietary intervention consisted of dietary counselling by a personal dietician coach (a trained dietetics student) to optimise their protein intake with the use of regular supermarket products. Contact visits were blended: face-to-face contacts and by tele-health contacts (primarily videoconferencing by Skype, or phone call when Skype failed). The dietary counselling targeted on a minimum of 1.2 g per kg body weight per day (g/kg BW/day) and the optimal amount of 1.5 g/kg BW/day. Subsequently, timing (breakfast, lunch, dinner, snacks, exercise), source of protein (high-quality protein sources, such as dairy protein), 25 g protein intake per meal, and personal factors (e.g., allergies, vegetarian pattern) were taken into account. Furthermore, the counselling was based on the Dutch Dietary guidelines 2015, including the introduction of nuts and leguminous vegetables and higher fish consumption [20]. Following standard practice, the dietician encouraged leaner meat consumption choices. The counselling was aiming for the continuation of grocery shopping in their preferred supermarket, at their own expense, and personalised to the participant. 

Directly after the baseline visit, the participants attended an educational group session of 45 min. The session aimed to develop first insight into the importance of proteins and available protein containing food products in supermarkets. Thereafter, the personalised coaching continued into the following order: week 1 videoconferencing; week 2 face-to-face group session at the community center; week 3 videoconferencing; week 4 monthly face-to-face group session at the community center; week 6–26 videoconferencing. Since the monthly face-to-face group sessions were scheduled for the exercise coach, the nutrition coach had an opportunity to set up a combined coach session. The major outline for the coaching period comprised weekly contact in the first two months, fortnightly in the next two months, and only once a month in the final two months. The coaching schedule is available in Supplementary Document S1. Dietary counselling was offered until 6 months; thereafter, the participants could continue their own preferred eating habits [16,19].

### 2.4. Dietary Behavior Change and Design Considerations

Three major functional design components of the dietary counselling intervention were (1) protein requirements, (2) behavior change, and (3) blended personalised counselling (videoconferencing and face-to-face coaching). The implementation of these components into the intervention and materials is available in Document S2.

### 2.5. Dietary Assessment

At all assessment visits, the 3-day dietary records were collected: the self-reported record contained 3 days, including 2 randomly chosen weekdays and 1 weekend day. Participants were able to fill out 6 eating occasions per day—breakfast, morning, lunch, afternoon, dinner, evening—representing the 3 main meals and 3 snack moments or smaller eating occasions. In addition, they were asked to report their food intake as specifically as possible, including standard household measures for amounts. If the records were incomplete, they were updated with the participant during their visit. Records were coded afterwards with the Dutch food composition table (NEVO, version 2013) and analysed with Amsterdam University of Applied Sciences (AUAS) developed syntax using SPSS package 25.0 (IBM, Armonk, NY, USA). Our laboratory developed an addition to the NEVO food composition table, including the estimation of the amino acid profiles. Participants with completed dietary records on at least two days were included for analysis. Additional verification and consistency check after coding is part of the standard operating procedure of our laboratory (e.g., energy intake of at least 800 kcal/day). Outcome was generated as macro and micronutrients per eating occasion, per day, and average of the three days. For protein intake, this resulted in the protein sources, product groups, resulting amino acid intake, and intake per eating occasion as an average of the three days.

### 2.6. Ethics

All junior coaches and students were intensively trained and supervised by the team of researchers and teachers of the AUAS. The research assistant was unblinded and therefore proceeded the randomisation with a computer-generated randomisation list. The study was approved by the Medical Ethics Committee (METC) of the VU University Medical Center (VUmc), The Netherlands (Protocol ID: VUMC2016_025) and registered at the NTR (NL5472). Written informed consent was obtained from all participants before inclusion.

### 2.7. Statistics

For all analyses, we used a linear mixed model (LMM) of repeated measures in order to adjust for the extra level of cluster and account for missing values in a clinical trial setting. Time and time*group interaction were defined as fixed factors, subject and cluster were included as random intercepts [21]. HBex-Pro was chosen as a reference group. For energy intake and protein outcome, age and gender were defined as potential confounders.

Changes in protein variables were visualised over the entire time course using the estimated marginal means. Within-group and intervention effects were reported as difference with 95% confidence interval and *p*-values. Statistical analyses were performed using SPSS Statistics v25.0 (IBM, Armonk, NY, USA) and the LMM with STATA/SE v13.0 (StataCorp LLC., College Station, TX, USA). An α of 0.05 was used to determine statistical significance.

## 3. Results

### 3.1. Study Participants

For this study, 224 randomised community-dwelling older adults of the randomised controlled trial (RCT) were assessed at the baseline visit [19]. Twelve dietary records were excluded from analysis due to missingness or invalidity. Dietary records of 212 participants were valid and available at baseline, 181 participants were available at 6 m, and 167 participants were available at 12 m; see the CONSORT flow chart at Figure S1. At baseline, the participants with an age (mean ± SD) of 72.2 ± 6.3 years had a BMI of 25.9 ± 4.2, an energy intake of 1880 ±4 72 kcal/day, protein intake of 1.08 ± 0.3 g/kg BW/day, and 44% were lower educated. Table 1 shows the baseline characteristics of the included cases of this analysis, with a focus on the nutritional status.

Overall, the groups were well balanced, and sexes were equally represented in each group. Differences in energy and protein intake (g/day, but not in g/kg BW/day) between male and female participants were noted as well as differences in muscle mass and strength between the sexes.

### 3.2. Digitally Supported Counselling—Coaching Contacts

To evaluate the dietary counselling, we analysed the different contact moments within a randomly selected group of participants (*n* = 52) who reached at least 5 m intervention (>80% completed). They experienced on average (median Inter Quartile Range [IQR]) 2.0 [1] physical contacts, (mean (SD)) 7.6 (1.8) tele-health/videoconferencing contacts and resulting in (median [IQR]) 10 [3] total dietician contacts.

### 3.3. Effectiveness of the Dietary Protein Counselling

#### 3.3.1. Macronutrients

Main changes in macronutrients were observed in protein intake after 6 m (+28.9 ± 2.8 g; *p* < 0.001) and 12 m (+15.9 ± 2.9 g; *p* < 0.001) in the dietary counselling group HBex-Pro. These participants increased their protein intake from 1.08 to 1.43 g/kg BW/day at 6 m and were able to sustain the intake up to 1.25 g/kg BW/day at 12 m. After the 6 m intervention, 72% of the participants in the HBex-Pro group, 35% in HBex, and 41% in CON achieved the daily protein intake target of 1.2 g/kg BW/day, and 42% in HBex-Pro, 13% in HBex, and 12% in CON achieved the target of 1.5 g/kg BW/day [19].

Total energy intake increased significantly in HBex-Pro after 6 m (+220 ± 60 kcal; *p* < 0.001) and decreased after the follow-up (+110 ± 62 kcal; *p* = 0.075). Fat consumption increased significantly only in the HBex-Pro group after 6 m (+11.6 ± 3.5 g; *p* = 0.001). The energy intake in kcal/day, but not kcal/kg BW/day, and fat intake in HBex-Pro at 6 m were significantly different from HBex and CON groups as shown as interaction effects. See Table S1.

#### 3.3.2. Protein Sources

**Figure 1:**
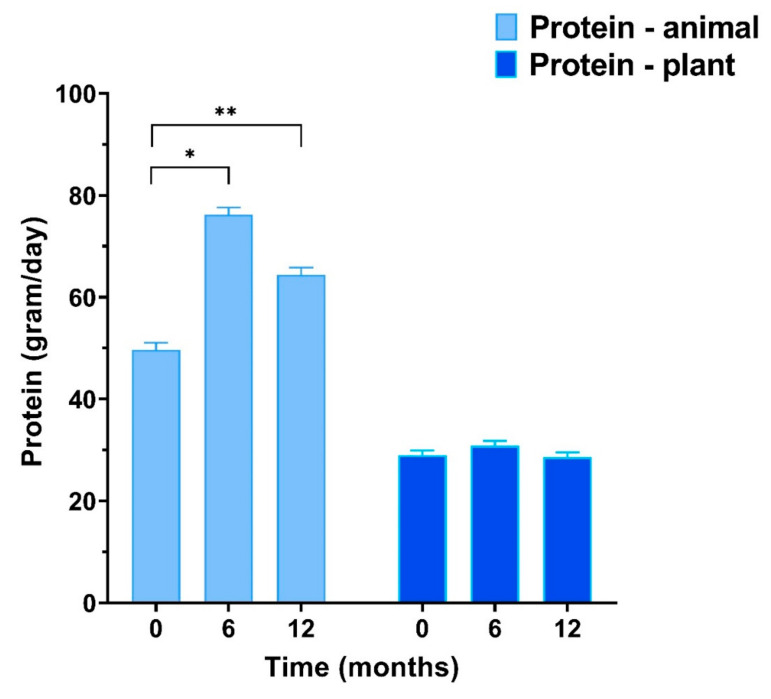
Protein intake from animal and plant-based protein sources at baseline, 6 months, and 12 months in HBex-Pro. Mixed models time effects (Difference ± SE): * +28.9 ± 2.8 *p* < 0.001, ** +15.9 ± 2.9 *p* < 0.001. Data are presented as estimated marginal means ± SEs.

Intake of protein from animal sources changed over the year due to the dietary counselling. After 6 m intervention, the animal protein intake in HBex-Pro increased from 49.7 ± 1.4 to 76.2 ± 1.4 g/day (6 m: +26.6 ± 2.6 g; *p* < 0.001) and maintained at 64.4 ± 1.4 g/day after the follow-up (12 m: +15.9 ± 2.9 g; *p* < 0.001), while the protein intake from plant sources remained unchanged. The increase in animal protein intake in the HBex-Pro group was significantly different compared to the HBex and CON groups, whereas they did not change in animal or plant protein intake (all interaction effects *p* < 0.001); see Table S1 and Figure 1.

#### 3.3.3. Product Groups

**Figure 2:**
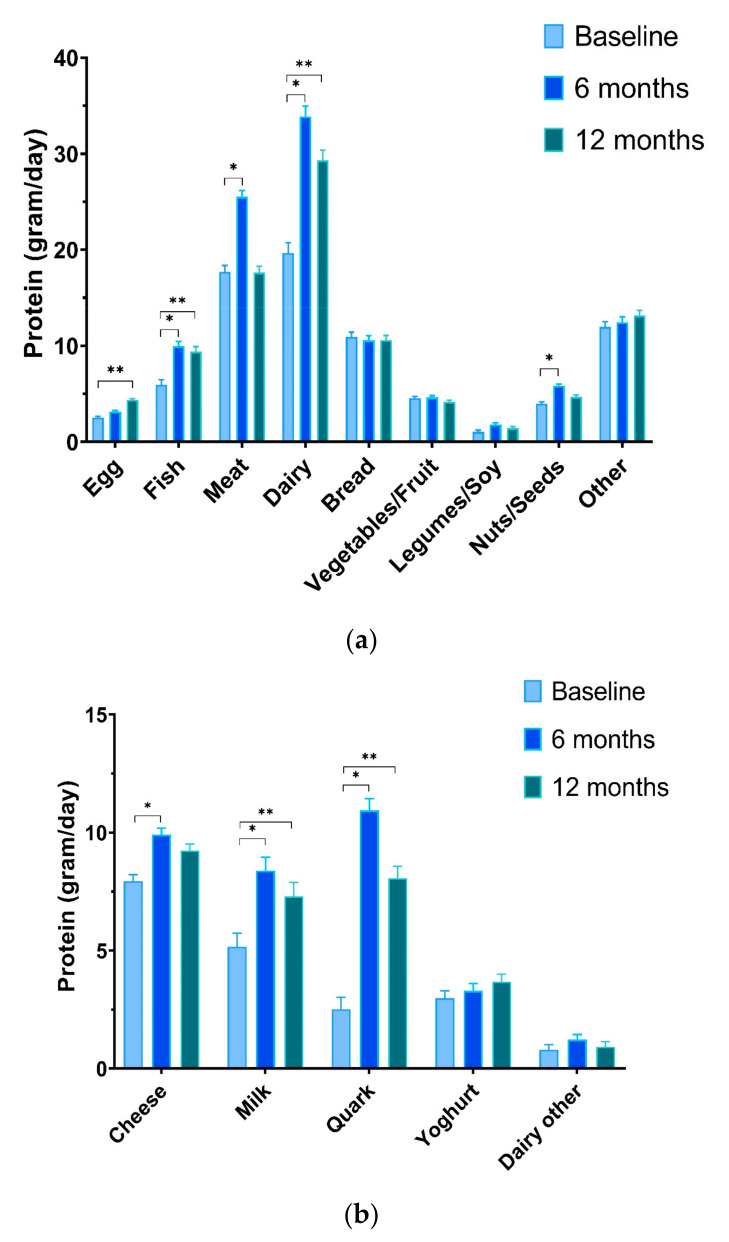
(**a**) Protein intake by product groups for HBex-Pro. Mixed models time effects (difference ± SE); Egg; ** +1.9 ± 0.4 *p* < 0.001; Fish; * +4.0 ± 1.3 *p* = 0.003|** +3.4 ± 1.4 *p* = 0.012; Meat; * +7.8 ± 2.1 *p* < 0.001; Dairy; * +14.2 ± 1.4 *p* < 0.001|** +9.7 ± 1.5 *p* < 0.001; Nuts/Seeds; ** +1.9 ± 0.7 *p* = 0.011; (**b**) Protein intake by dairy product groups for HBex-Pro. Cheese; * +2.0 ± 0.9 *p* = 0.034; Milk; * +3.2 ± 0.7 *p* < 0.001|** +2.1 ± 0.7 *p* = 0.002; Quark; * +8.4 ± 0.8 *p* < 0.001|** +5.6 ± 0.8 *p* < 0.001; Data are presented as estimated marginal means ± SEs.

Significant changes in protein intake from the product groups egg, fish, meat, dairy, and nuts/seeds were observed in the HBex-Pro group only. Intake of egg protein increased from 2.5 ± 0.1 g/day to 3.1 ± 0.1 g/day at 6 m and 4.4 ± 0.1 g/day at 12 m (*p* < 0.001). Fish protein intake increased after the dietary counselling (6 m: +4.0 ± 1.3 g; *p* = 0.003) and after follow-up (12 m: +3.4 ± 1.4 g; *p* = 0.012), and it was significantly higher compared to both other groups. Intake of meat protein was significantly increased in the HBex-Pro group only at 6 m (+7.8 ± 2.1 g; *p* < 0.001), and it returned back to baseline levels after the follow-up. The increase at 6 m was not significantly different compared to the meat protein intake in the HBex and CON groups. Protein intake from dairy products accounted for a large increase in HBex-Pro from 19.7 ± 1.1 g/day to 33.9 ± 1.1 g/day at 6 m and 29.3 ± 1.1 g/day at 12 m. These increases (6 m; +14.2 ± 1.4 g; *p* < 0.001; 12 m +9.7 ± 1.5 g; *p* < 0.001) were significantly higher than the other two groups (all interaction effects *p* < 0.001); see Table S1 and Figure 2a. Protein intake from nuts and seeds was higher at 6 m (+1.9 ± 0.7 g; *p* = 0.011), which is significantly different from the other two groups, but it decreased after follow-up.

Further analysis of the dairy intake revealed that quark, milk, and cheese products significantly contributed to the increased protein intake in the HBex-Pro group. Quark products showed an increase at 6 m of +8.4 ± 0.8 g (*p* < 0.001) and remained higher after follow-up (12 m: +5.6 ± 0.8; *p* < 0.001). Milk protein consumption also increased (6 m: +3.2 ± 0.7 g; *p* < 0.001; 12 m: +2.1 ± 0.7 g; *p* = 0.002) and both quark and milk showed significant intervention effects compared to the two other groups. Protein intake from cheese products were significantly increased in HBex-Pro at 6 m (+2.0 ± 0.9 g; *p* = 0.034), as well as compared to HBex (*p* = 0.004). See Table S1 and Figure 2b.

#### 3.3.4. Intake of Amino Acids

All amino acid categories, essential and non-essential, branched chain amino acids (BCAA) and leucine, showed significant increased levels both within HBex-Pro and compared to HBex and CON groups. Intake of essential amino acids (6 m: +12.0 ± 1.2 g; *p* < 0.001; 12 m: +5.8 ± 1.2 g; *p* < 0.001) and non-essential amino acids (6 m: +11.8 ± 1.3 g; *p* < 0.001; 12 m: +5.1 ± 1.4 g; *p* < 0.001) were both increased in HBex-Pro as well as BCAA intake (6 m: +5.4 ± 0.5 g; *p* < 0.001; 12 m:+2.7 ± 0.6 g; *p* < 0.001). Leucine intake was 2.4 ± 0.2 g (*p* < 0.001) higher in the HBex-Pro group at 6 m and +1.2 ± 0.2 g (*p* < 0.001) higher at 12 m; see Table S1.

**Figure 3:**
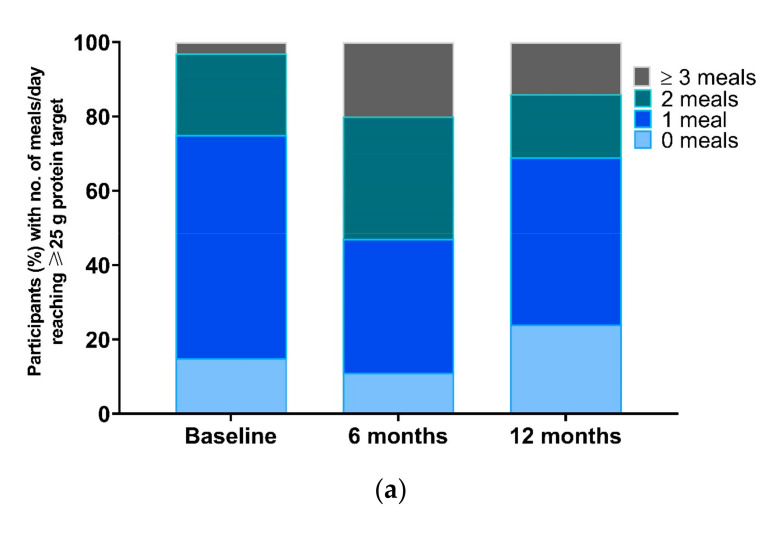

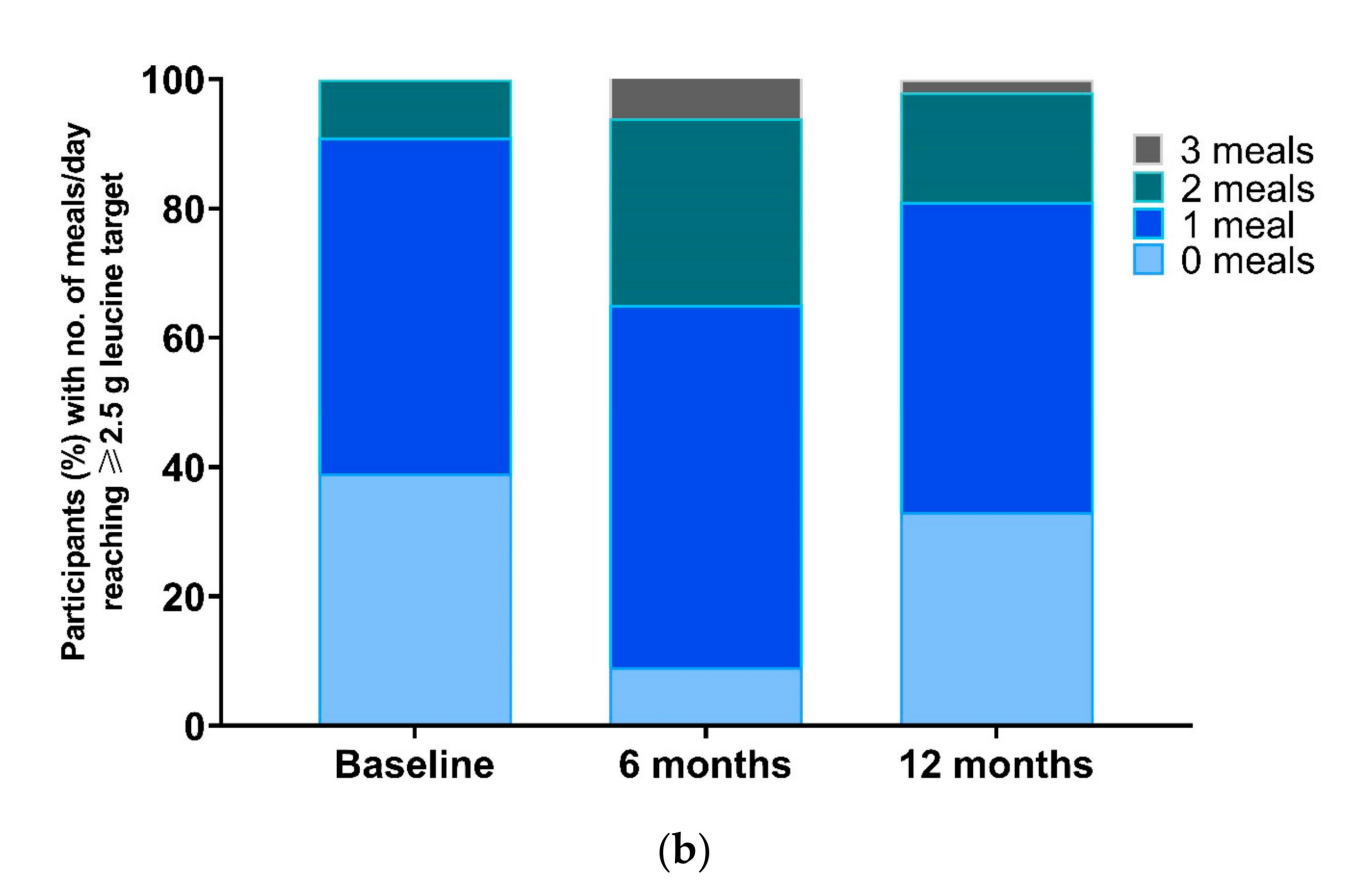
Percentages of participants in the HBex-Pro group at baseline, 6 months, and 12 months consuming no, one, two, or three meals per day, reaching the targets of (**a**) ≥25 g protein per meal; or (**b**) the ≥2.5 g leucine target per meal.

The effect of dietary counselling with respect to the leucine target of providing ≥2.5 g/meal is shown in Figure 3b and Table S2. After 6 m intervention, the percentage of participants in the HBex-Pro group that were not able to consume any meal at the leucine target decreased from 39% to 9%, while the percentage that consumed on average two or more meals per day reaching the leucine target increased from 9% to 36%. These percentages decline at the 6 m follow-up, but in comparison to the other two groups, the percentage of the group with no meals reaching the leucine target was still lower in the HBex-Pro group. In addition, the percentage of this group consuming two or three meals providing ≥2.5 g leucine was higher compared to the other two groups.

#### 3.3.5. Protein Intake at Different Eating Occasions

The increase of protein intake in the HBex-Pro group was distributed evenly over the day with major changes at breakfast and lunch. Protein intake at breakfast after the 6 m personalised intervention increased significantly (6 m: +6.2 ± 1.0 g; *p* < 0.001) and remained at that level during the follow up (12 m: +6.7 ± 1.1 g; *p* < 0.001). Participants in HBex-Pro also consumed more protein at lunch (6 m: 7.5 ± 1.2 g; *p* < 0.001; 12 m +4.2 ± 1.2 g; *p* = 0.001), and after 6 m, the mean protein intake at lunch reached the target of 25 g per meal (25.8 ± 0.7 g). Dinner showed a significant increased level at 6 m only (+10.6 ± 1.9 g; *p* < 0.001) and returned to baseline levels after the follow up.

**Figure 4:**
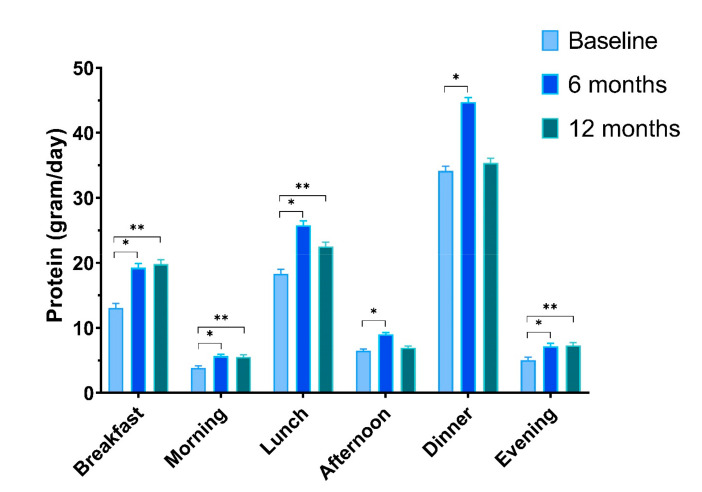
Protein intake at eating occasions at baseline, at 6 and 12 months for HBex-Pro. Mixed models time effects (Difference ± SE): Breakfast; * +6.2 ± 1.0 *p* < 0.001|** +6.7 ± 1.1 *p* < 0.001; Morning; * +1.8 ± 0.7 *p* = 0.007|** +1.7 ± 0.7 *p* = 0.012; Lunch; * +7.5 ± 1.2 *p* < 0.001|** +4.2 ± 1.2 *p* = 0.001; Afternoon; * +2.5 ± 1.0 *p* = 0.009; Dinner; * +10.6 ± 1.9 *p* < 0.001; Evening; * +2.1 ± 0.7 *p* = 0.004|** +2.3 ± 0.7 *p* = 0.003. Data are presented as estimated marginal means ± SEs.

Regarding the snack moments or smaller eating occasions, the morning (6 m +1.8 ± 0.7 g; *p* = 0.007; 12 m +1.7 ± 0.7 g; *p* = 0.012) and evening (6 m +2.1 ± 0.7 g; *p* = 0.004; 12 m +2.3 ± 0.7 g; *p* = 0.003) showed effects after the dietary counselling and follow up. The afternoon snack moment showed only increased protein intake at 6 m (+2.5 ± 1.0 g; *p* = 0.009). Most effects seen in HBex-Pro correspond with interaction effects between the groups; see Table S1 and Figure 4.

The effect of dietary counselling with respect to the protein target of providing ≥25 g/meal is shown in Figure 3a and Table S3. After 6 m intervention, the percentage of the group consuming two or three compliant meals increased from 22% to 33% and 3% to 20% respectively, which was mainly by increasing protein intake during breakfast and lunch. The percentage of participants in the HBex-Pro group that consumed no or only one meal that reached the protein target decreased from 15% to 11% and 60% to 36%, respectively. After 6 m follow-up, the percentages on two or three meals in HBex-Pro decline, although 14% is maintained for more than three meals and higher than before the intervention.

## 4. Discussion

The combined exercise and nutritional intervention including digitally supported dietary counselling showed to be effective in supporting the community-dwelling older participants to increase their dietary protein intake in the range from 1.0 to 1.5 g/kg BW/day as recommended for older adults [11,12]. The older adults in this intervention consumed more protein from animal sources, particularly dairy products, meat, and fish. This also resulted in more essential amino acid intake, including more meals providing at least 2.5 g of leucine. The protein intake was distributed evenly over the day, mainly by increasing protein intake during breakfast and lunch, resulting in more meals providing at least 25 g protein.

Although the dietary counselling was based on advice on both animal and plant-based protein sources, the main significant increases in intake were found in animal protein sources. Only a small increase in plant protein intake from nuts and seeds was observed, which are protein sources that are also recommended by the National Dietary Guidelines [20]. Since animal proteins have a higher quality than plant-based proteins based on the DIAAS values [15], the blended intervention may have enhanced both the quantity and the quality of the protein intake. The improved quality of the protein intake is even more relevant when the protein intake is limited in older adults, which is often due to reduced appetite [13].

Further classification in product groups revealed that the major part of the increased protein intake was consistently derived from dairy product groups. The consumption of dairy products is recommended by the National Dietary guideline [20] and thus also part of the dietary counselling. The increase in dairy product intake was due to more consumption of quark, milk, and cheese. These products were suitable to maintain a change in eating behavior up to 12 months, which is in line with previous studies that show that dairy proteins are well tolerated in older adults [23]. This suggests that dairy products, especially high protein products such as quark with ≈25 g protein/250 g serving, are feasible products to increase the intake of high-quality protein consistently in this population. Meat and fish were two other product groups that contributed to the increase of animal protein intake. The consumption of meat increased after 6 months, but it decreased back to baseline levels after 12 months. The increase of meat consumption is not part of current guidelines; in fact, lower meat consumption is encouraged for sustainability reasons. The increase of meat consumption may also have contributed to the increased energy and fat intake at 6 months in this group. These participants also consumed more fish, as is recommended by the dietary guidelines. Most older adults actually replaced their meat consumption once or twice weekly by fish and discovered a new product group to adapt a healthier diet.

The amount of protein intake per eating occasion, the quality of ingested protein, and the distribution over the day are important determinants to maximally stimulate muscle protein synthetic response and thus support muscle preservation. The recommendations for older adults to consume 25–30 g of a high-quality protein per meal, providing at least 2.5 g leucine, and the meals evenly distributed throughout the day [24,25,26] were targets for the nutritional intervention. The observed increase of protein intake due to the digitally supported dietary counselling of the community dwelling older participants was indeed evenly distributed throughout the day. The percentage of older adults consuming two or three meals providing 25 g protein increased after the 6 months intervention, mainly by increasing the protein intake at breakfast and lunch. A part of the study population maintained to consume two or three meals at the protein target after the follow-up, showing the challenge to reach this target without intensive support. Although the advised 25 g protein per main meal was on average not reached, the average intake towards 20 g protein at breakfast and 20–25 g at lunch was achievable and sustainable with regular food products from the supermarket in this population of community-dwelling older adults. In line with the increased consumption of high-quality protein sources, the total intake of all essential amino acids including leucine was elevated in the dietary counselling group.

Those participants also consumed more meals providing the recommended 2.5 g leucine in order to overcome the leucine threshold for the stimulation of muscle protein synthesis [27]. The increase of percentage of participants that consumed two or three meals reaching the protein and leucine targets shows the ability to introduce a second and third meal with sufficient protein and leucine in addition to dinner, which is often already rich in high-quality protein and thus leucine. The improved protein and leucine intake per meal distributed over the day might have contributed to the effects in muscle functioning outcomes in the HBex-Pro group compared to the other groups [19].

Dietary counselling on protein intake has shown to be effective in several populations of older adults [28,29,30,31,32]. Comparable studies about digitally supported dietary counselling (using e-health) targeting on increased protein intake with supermarket products are lacking in community-dwelling older adults. Meanwhile, digital support as part of nutritional counselling has been introduced in populations with chronic diseases and illness [33,34,35]. As well, the application of e-health in nutritional interventions is growing [36].

Our digitally supported dietary counselling was designed to contribute to dietary behavior change in protein intake, and it was offered in addition to a home-based exercise program. Both interventions incorporated similar effective behavior change techniques; e.g., goal setting, self-monitoring, motivational interviewing, and feedback [37,38,39]. Although the different aspects of the intervention were offered by separate professionals, the interventions were designed to complement each other with a corresponding behavior change framework.

Important beneficial aspects of digitally supported dietary counselling for community-dwelling older adults are the access to the health care provider, reduced coping with travel limitations for face-to-face consultations [40], and cost-effectiveness for dietetics practice [41]. Due to the combination of group contacts and individual counselling contacts, several supportive techniques related to behavior change are targeted. The benefits of using social support, direct experience, feedback, coping, and learning from peers along the group setting, in combination with the self-regulatory techniques in the individual setting, have been combined [37,42,43]. Nowadays, this combination of group and individual contacts is widely adapted to effective lifestyle interventions, especially interventions that target exercise and nutritional behavior change for obesity and/or diabetes [44,45].

E-health supported interventions in older adults are not commonly used in practice. One reason for lacking interventions on blended or digitally supported counselling on nutrition in older adults is the lacking e-health literacy in older adults [46] and possibly the dietician (health care provider). Recent evidence shows that a large amount of interventions do not encounter e-health literacy [47]. Our experience shows that self-efficacy and competence in e-health use can be increased within this population by incorporating time to adapt to the electronic device. Even older adults with no previous device experience were able to participate [18,48]. Our systematically designed intervention is a major strength, because of engagement of the end user, and pilots have been performed before the trial. The novel blended approach including personalised counselling with regular supermarket products is a pragmatic approach for the feasible and affordable implementation in daily life and dietetic practice.

The generalisability of these results is subject to certain limitations. As this dietary behavior change intervention was designed for the Dutch population, including the National Dietary Guidelines, the design of our intervention and the results should be generalised with caution for all community-dwelling older adults. The population is community-dwelling with a relative healthy lifestyle. Therefore, implementation of the results to all community-dwelling older adults, including the more frail population, needs further investigation. Nevertheless, the results on diet adaptations in protein intake are supportive for further exploration in the diverse population of community-dwelling adults and supportive for future dietetics practice.

## 5. Conclusions

Blended, digitally supported dietary counselling was effective in increasing protein intake in a lifestyle intervention in community-dwelling older adults. The increased protein intake was predominantly achieved by consuming more animal protein sources, particularly dairy products. This also resulted in more essential amino acid intake, including more meals providing at least 2.5 g of leucine. The protein intake was distributed evenly over the day, mainly by increasing protein intake during breakfast and lunch, resulting in more meals providing at least 25 g protein. This blended approach of digitally supported dietary counselling as part of a lifestyle intervention is promising for large-scale implementation by dieticians in the population of community-dwelling older adults.

## Figures and Tables

**Table 1:** Baseline characteristics for participants.

Characteristics *		Total (*n* = 212)	CON (*n* = 84)	HBex (*n* = 63)	HBex-Pro (*n* = 65)
Age (years)		72.2 (6.3)	73.0 (6.5)	72.3 (5.5)	71.0 (6.6)
Female sex *n* (%)		148 (70)	59 (70)	42 (67)	47 (72)
Level of education ^a^	Low education *n* (%)	93 (44)	38 (45)	27 (43)	28 (43)
Ethnicity	Caucasian *n* (%)	201 (95)	81 (96)	59 (94)	61 (94)
MMSE score		28.3 (2.0)	28.3 (1.6)	28.6 (1.7)	27.9 (2.6)
Weight	kg	73.6 (13.9)	73.3 (12.7)	72.4 (14.4)	75.1 (14.9)
BMI	kg/m^2^	25.9 (4.2)	25.7 (3.5)	25.3 (3.9)	26.8 (5.1)
Sarcopenia ^b^ *n* (%)	No Sarcopenia	196 (92)	79 (94)	59 (94)	58 (89)
	Probable	14 (6)	4 (5)	4 (6)	6 (9)
	Confirmed	2 (1)	1 (1)	0 (0)	1 (2)
Frailty score ^c^ *n* (%)	Non-Frail	185 (87)	72 (86)	58 (92)	55 (85)
	Mildly Frail	24 (11)	12 (14)	3 (5)	9 (16)
	Moderately Frail/Frail	3 (1)	0 (0)	2 (3)	1 (2)
Medical conditions ^d^ *n* (%)	Musculoskeletal	125 (59)	49 (58)	39 (62)	37 (57)
	Arthrosis	49 (23)	16 (19)	15 (23)	18 (28)
	Cardiovascular	115 (54)	50 (58)	33 (52)	32 (49)
	Orthopedic implants	27 (13)	8 (10)	12 (19)	7 (11)
	Respiratory	18 (8)	4 (5)	7 (11)	7 (11)
	Diabetes type II	13 (6)	3 (4)	5 (8)	5 (8)
	Comorbidity(≥2 diseases)	117 (55)	47 (56)	35 (56)	35 (54)
					
*Nutritional intake—Macronutrients*					
Energy	kcal/day	1880 (472)	1899 (450)	1817 (433)	1918 (533)
	kcal/kg/day	26.2 (7.6)	26.3 (6.4)	26.2 (8.5)	26.1 (8.1)
Carbohydrates	g/day	186 (55.7)	186 (55.5)	185 (51.9)	188 (60.2)
Fat	g/day	75.4 (25.3)	77.5 (23.6)	71.7 (22.4)	76.3 (29.7)
Protein	g/day	77.8 (20.1)	78.8 (20.1)	74.6 (19.2)	79.4 (20.8)
	g/kg/day	1.08 (0.3)	1.09 (0.26)	1.06 (0.30)	1.07 (0.31)
Protein—Animal source	g/day	49.3 (17.1)	50.4 (18.5)	46.9 (16.8)	50.2 (15.7)
Protein—Plant source	g/day	28.5 (8.8)	28.3 (8.1)	27.8 (8.5)	29.3 (10.0)

* Reported as means (SD) unless stated otherwise. CON, Control group; HBex, Home-based exercise training group; HBex-Pro, Home-based exercise training with dietary protein counselling group. MMSE, mini mental state examination; ^a^ Low education defined as community college or less educated (primary and secondary education). ^b^ Sarcopenia score derived from Cruz-Jentoft 2019 [22]; based on HGS, ALM/height^2^ and gait speed. ^c^ Frailty score derived from m-PPT score. ^d^ Disease categories based on ICD-10.

## Data Availability

The data presented in this study are available on request from the corresponding author.

## Supplementary Materials

The following are available online at https://www.mdpi.com/2072-6643/13/2/502/s1, Document S1: Coaching schedule of the interventions, Document S2: Dietary counselling, design, materials and BCTs, Figure S1: CONSORT flow chart, Table S1: Effects of protein counselling on protein intake in older adults, Table S2: Frequencies of leucine compliers per meal (≥2.5 g) for all groups, Table S3: Frequencies of protein compliers per meal (≥25 g) for all groups.
