# Supplementary material for: Digitally Supported Dietary Protein Counseling Changes Dietary Protein Intake, Sources, and Distribution in Community-Dwelling Older Adults
Source: Nutrients. 2021 Feb 3;13(2):502. doi: 10.3390/nu13020502 (PMC7913661; doi:10.3390/nu13020502)
Supplement: Supplementary file 1 [file nutrients-13-00502-s001.pdf]

Supplementary Document S1. Coaching schedule of the interventions

Article title: Digitally supported dietary protein counselling changes dietary protein intake, sources and distribution in community-dwelling older adults

Journal name: Nutrients

Author names: Jantine van den Helder, Sjors Verlaan, Michael Tieland, Jorinde Scholten, Sumit Mehra, Bart Visser, Ben J.A. Kröse, Raoul H.H. Engelbert and Peter J.M. Weijs.

Corresponding Author: Jantine van den Helder, Faculty of Sports and Nutrition, Center of Expertise Urban Vitality, Amsterdam University of Applied Sciences, Dokter Meurerlaan 8, 1067 SM, Amsterdam, the Netherlands
T +31621155919 | Email j.e.m.van.den.helder@hva.nl

Coaching schedule for 6-month intervention period

Week	Activity	Exercise coach	Diet coach
**0**	Baseline visit (0 months)	Phone call for appointment	Group education session (60min)<br>Introduction and home material
**1**	Introduction + tablet PC and home package	F2F - Home visit (90min)	Phone call or VC (15min)
**2**	Goal-setting (exercise)<br>Group evaluation (diet)	F2F - Home visit (90min)	F2F - Community center (60min)
**3**	Coach session	VC (15min)	VC (15min)
**4**	Coach session	VC (15min)	VC (15min)
**5**	Monthly group consultation	F2F - Community center (60min)	F2F - Community center (together)
**6**	Coach session	F2F - Home visit (60min)	VC (15min)
**7**	Coach session	VC (15min)	-
**8**	Coach session	VC (15min)	VC (15min)
**9**	Monthly group consultation	F2F - Community center (60min)	If desired - F2F with exercise coach
**10**	Adjusting goals<br>Coach session	F2F - Home visit (60min)	VC (15min)
**11**	-	-	-
**12**	Coach session	VC (15min)	VC (15min)
**13**	Monthly group consultation	F2F - Community center (60min)	-
**14**	Coach session	F2F - Home visit (45min)	VC (15min)
**15**	-	-	-
**16**	Coach session	VC (15min)	VC (15min)
**17**	Monthly group consultation	F2F - Community center (60min)	If desired - F2F with exercise coach
**18**	Coach session	VC (15min)	-
**19**	-	-	-
**20**	Coach session	-	VC (15min)
**21**	Monthly group consultation	F2F - Community center (60min)	If desired - F2F with exercise coach
**22**	Coach session<br>(operating independently)	F2F - Home visit (45min)	-
**23**	-	-	-
**24**	Coach session	-	VC (15min)
**25**	Monthly group consultation<br>Closing session(s)	F2F - Community center (60min)	F2F - Community center
**26**	Effect visit (6 months)		

This overview presents the contacts for the exercise and diet coaches, including the prescribed time. Detailed description of the coaching intervention was available in the coach manual. F2F, face-to-face contact; VC, Video call with Skype.

Supplementary Document S2. Dietary counselling, design, materials and BCTs

Article title: Digitally supported dietary protein counselling changes dietary protein intake, sources and distribution in community-dwelling older adults

Journal name: Nutrients

Author names: Jantine van den Helder, Sjors Verlaan, Michael Tieland, Jorinde Scholten, Sumit Mehra, Bart Visser, Ben J.A. Kröse, Raoul H.H. Engelbert and Peter J.M. Weijs.

Corresponding Author: Jantine van den Helder, Faculty of Sports and Nutrition, Center of Expertise Urban Vitality, Amsterdam University of Applied Sciences, Dokter Meurerlaan 8, 1067 SM, Amsterdam, the Netherlands
T +31621155919 | Email j.e.m.van.den.helder@hva.nl

Dietary counselling, design, materials and BCTs

The dietary protein counselling intervention is developed with a similar approach as the blended home-based exercise program [17]. The process of the development of the design, feasibility phase until this Evaluation study is described following the Medical Research Council (MRC) framework for complex interventions. The combination of scientific literature, as well as practice-based evidence was encountered.

Development
> Identifying the evidence base / modeling process and outcomes

Feasibility and Piloting
> Testing procedures

Evaluation
> RCT: Assessing effectiveness

Development

Design considerations

Blended HB-exercise intervention	Dietary protein counselling intervention
1) Functional exercises<br>2) Behavior change<br>3) Blended technology	1) Protein requirements<br>2) Behavior change<br>3) Blended counselling

Dietary protein counselling intervention

1) Protein requirements

As described in this paper, the previous published protocol paper[16] and effect paper[19]:

- Goal: a minimum of 1.2 g per kg body weight per day (g/kg BW/day), and the optimal amount of 1.5 g/kg BW/day

Subsequently:

- Timing: breakfast, lunch, dinner, snacks, exercise (eating occasions)
- Source of protein: high quality protein sources, such as dairy protein
- Amount per meal: 25 g protein per meal
- Personalization: personal factors (e.g. allergies, vegetarian pattern)
- Healthy eating pattern: Dutch Dietary guidelines 2015
- Adaptability: grocery shopping in supermarket of own choice & own expense

2) Behavior change

In our previous paper of the design of the blended intervention [17], the behavior change technique (BCT) taxonomy is introduced [43]. Techniques that are associated with the self-regulation of behavior appear particularly effective: goal-setting and self-monitoring [37]. In addition, other techniques that increase self-efficacy were added to encounter for effective strategies in the blended counselling. The self-determination theory (SDT) is a theoretical framework of human motivation and behavior developed by Deci and Ryan[1]. The SDT states that an autonomous style of self-regulation (identified, integrated and intrinsic) leads to more positive behavioral outcomes. For individual dietary counselling Motivational Interviewing (MI) is chosen as intervention technique, as it shares the same values as SDT [39].

Within behavioral nutrition and exercise interventions that target of lifestyle changes, the combination of group contacts with individual contacts is seen more often as successful [44,45]. The opportunity arises to incorporate several group-related BCTs and individual-related BCTs into the intervention components and materials.

3) Blended counselling

Our blended counselling can be introduced as the combination of face-to-face contacts and tele-health contacts (primarily videoconferencing with an application, as mentioned as digitally supported). Digitally supported dietary counselling has benefits of remote guidance including non-verbal communication, reduced travel time and costs. In current dietetics practice face-to-face contacts are most often used, which is beneficial for the clients' trust and to exchange documents. Especially in the population of older adults, the face-to-face contacts are common practice.

Identifications of the requirements for the dietician coach

Coaching tools

1) Feedback and monitoring

2) Interprofessional communication

3) Reporting

> A shared coaching manual was developed and a coach CMS website. The coaching manual included information on the Coaching schedule (See **Additional Document S1**), theoretical framework, goals per visit, and other features to be able to carry out the intervention. We were aiming to improve knowledge, competence and skills, in order to increase the level of expertise of the (student) coaching professional. Topics as e-coaching, motivational interviewing and interprofessional collaboration were included. The coach CMS website was designed for interprofessional communication and reporting purposes.

Dietary counselling materials and BCTs

Additional to the scientific evidence, the requirements and functional components were collected by use of expert interviews. The following intervention materials were developed or encountered.

**Functional component of the Dietary protein counselling intervention**	**BCTs**
Videoconferencing app (Skype)	behavioral rehearsal, graded tasks, verbal persuasion about capability.
Classroom lecture at baseline assessment; elaboration session	information about health consequences, framing/reframing, behavioral practice/rehearsal (workbook/reading food labels), guided practice (direct experience/tasting), goal setting (1.2–1.5 g/kg/day), social comparison.
Information magazine with emphasis on protein	rehearsal of information (knowledge/competence), behavioral rehearsal.
Two-week workbook; including recipes, protein product group list	self-monitoring of behavior & goals, habit formation, behavior rehearsal, action planning.
Group session after two weeks	guided practice (videoconferencing/increase e-health literacy), feedback on behavior (coach/workbook), goal setting, graded tasks, problem solving, planning coping responses, social support (practical/emotional), social reward, verbal persuasion about capability.
Monthly group visit	e.g. action planning, problem solving, planning coping responses, social support.

Individual video-conferencing and counselling	social support, social incentive/reward, action planning, feedback on behavior, goal evaluation, prompts/cues, problem solving (motivational interviewing).

Feasibility and Piloting

A pilot was performed with the dietary counselling intervention and materials with three existing exercise groups of community-dwelling older adults. These were selected from another region in the Netherlands, as the target population of the RCT was the Amsterdam metropolitan region.
> After the pilots the information brochure and coaching manual was further developed and improved.

Evaluation
> RCT: Assessing effectiveness. The results of this dietary protein counselling intervention is furthermore published in this article.

Reference:
[1] Deci EL, Ryan RM: The "What" and "Why" of Goal Pursuits: Human Needs and the Self Determination of Behavior. Psychological Inquiry 2000, 11: 227-268.

Supplementary Figure S1. CONSORT flow chart

Article title: Digitally supported dietary protein counselling changes dietary protein intake, sources and distribution in community-dwelling older adults

Journal name: Nutrients

Author names: Jantine van den Helder, Sjors Verlaan, Michael Tieland, Jorinde Scholten, Sumit Mehra, Bart Visser, Ben J.A. Kröse, Raoul H.H. Engelbert and Peter J.M. Weijs.

Corresponding Author: Jantine van den Helder, Faculty of Sports and Nutrition, Center of Expertise Urban Vitality, Amsterdam University of Applied Sciences, Dokter Meurerlaan 8, 1067 SM, Amsterdam, the Netherlands

T +31621155919 | Email j.e.m.van.den.helder@hva.nl

Figure S1. CONSORT flow diagram of VITAMIN study clusters and participants. IQR = interquartile

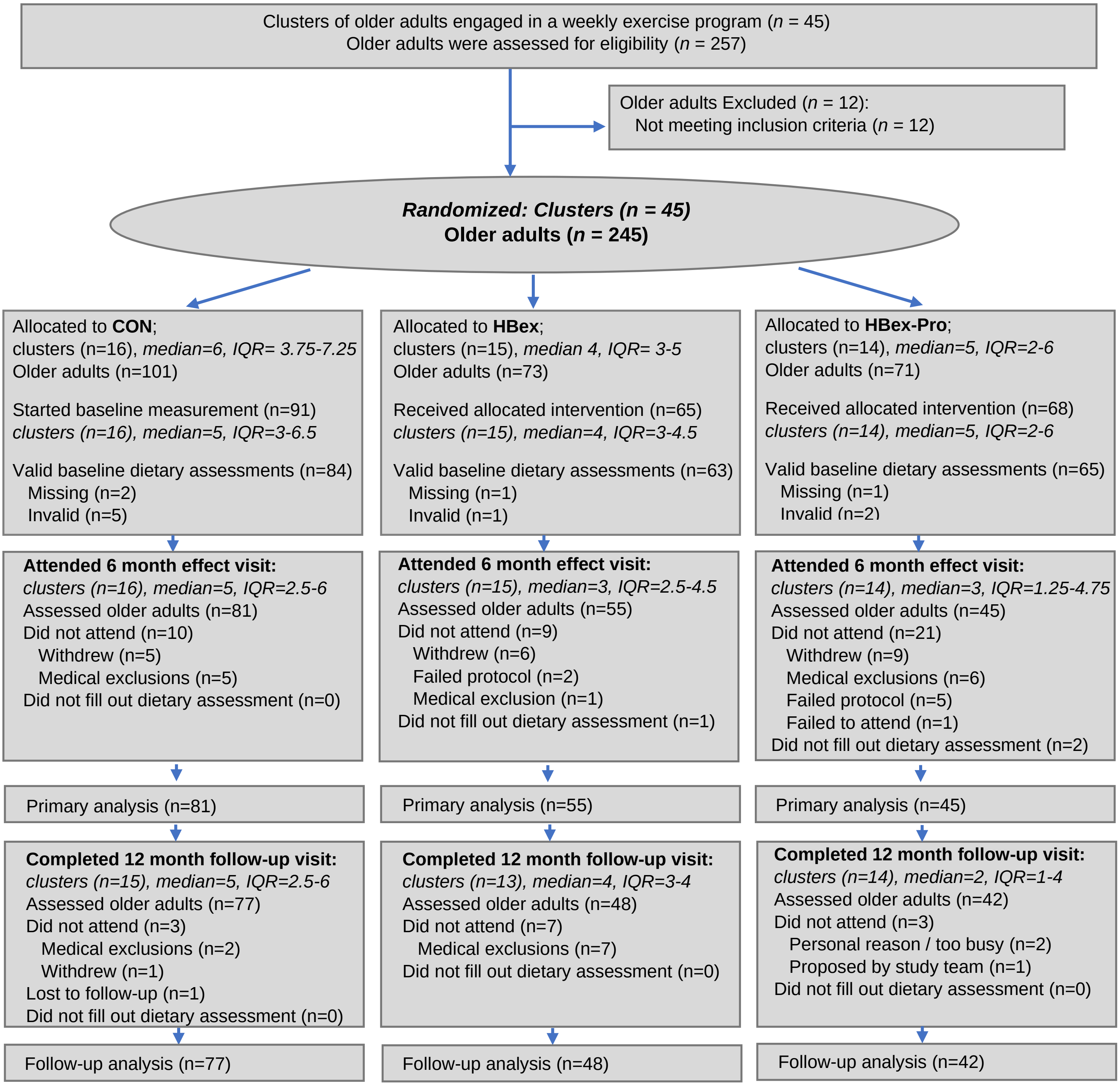

Supplementary TableS1. Effects of protein counselling on protein intake in older adults

Article title: Digitally supported dietary protein counselling changes dietary protein intake, sources and distribution in community-dwelling older adults

Journal name: Nutrients

Author names: Jantine van den Helder, Sjors Verlaan, Michael Tieland, Jorinde Scholten, Sumit Mehra, Bart Visser, Ben J.A. Kröse, Raoul H.H. Engelbert and Peter J.M. Weijs.

Corresponding Author: Jantine van den Helder, Faculty of Sports and Nutrition, Center of Expertise Urban Vitality, Amsterdam University of Applied Sciences, Dokter Meurerlaan 8, 1067 SM, Amsterdam, the Netherlands
T +31621155919 | Email j.e.m.van.den.helder@hva.nl

Table S1. Effects of protein counselling on protein intake in older adults. Values are estimated marginal means (SE) unless stated otherwise										
		**Randomized groups**			**MM time effects**	**MM interaction effects***				
**Outcome variable**		**HBex-PRO #**	**HBex**	**CON**			**HBex-PRO vs HBex †**		**HBex-PRO vs CON ‡**	
		EMM (SE)	EMM (SE)	EMM (SE)	Difference (SE) *p*-value		Difference (SE 95%CI)	P value	Difference (95%CI)	P value
Energy & Macro nutrients										
Energy[a]	0 m	1900 (42)	1851 (37)	1887 (33)						
(Kcal/day)	6 m	2119 (42)	1862 (37)	1930 (33)	**+220 (60) *p*<0.001**	Intervention effect	−208.0 (−359.7;−56.2)	**0.007**	−176.8 (−319.8;−33.8)	**0.015**
	12 m	2009 (42)	1863 (37)	1860 (33)	+110 (62) *p*=0.075	Follow-up effect	−97.2 (−255.8;61.4)	0.230	−136.8 (−283.7;10.1)	*0.068*
Energy	0 m	26.1 (0.7)	26.2 (0.9)	26.3 (0.5)						
(Kcal/kg/day)	6 m	28.1 (0.7)	26.9 (0.9)	27.1 (0.5)	**+1.98 (0.9) *p*=0.028**	Intervention effect	−1.20 (−3.51;1.10)	0.306	−1.18 (−3.35;0.98)	0.284
	12 m	26.6 (0.7)	26.9 (0.9)	26.0 (0.5)	+0.51 (0.9) *p*=0.579	Follow-up effect	+0.19 (−2.21;2.60)	0.875	−0.83 (−3.05;1.39)	0.464
Carbohydrates	0 m	187.0 (5.4)	185.1 (4.3)	186.0 (4.0)						
(g/day)	6 m	192.8 (5.4)	187.4 (4.3)	189.9 (4.0)	+5.79 (6.6) *p*=0.382	Intervention effect	−3.57 (−20.5;13.4)	0.680	−1.88 (−17.8;14.0)	0.817
	12 m	191.5 (5.4)	189.1 (4.3)	185.8 (4.0)	+4.54 (6.8) *p*=0.506	Follow-up effect	−0.57 (−18.3;17.1)	0.949	−4.71 (−21.1;11.6)	0.572
Fat	0 m	75.8 (1.9)	73.7 (1.6)	76.4 (1.4)						
(g/day)	6 m	87.3 (1.9)	73.6 (1.6)	79.4 (1.4)	**+11.6 (3.5) *p*=0.001**	Intervention effect	−11.68 (−20.5;−2.82)	**0.010**	−8.62 (−16.9;-.29)	**0.043**
	12 m	80.8 (1.9)	75.1 (1.6)	75.7 (1.4)	+4.99 (3.6) *p*=0.168	Follow-up effect	−3.58 (−12.8;5.7)	0.450	−5.69 (−14.2;2.87)	0.193
Protein	0 m	78.6 (1.8)	76.1 (1.6)	78.3 (1.3)						
(g/day)	6 m	107.4 (1.8)	78.1 (1.6)	81.1 (1.3)	**+28.9 (2.8) *p*<0.001**	Intervention effect	−26.9 (−34.0;−19.7)	**<0.001**	−26.2 (−32.9;−19.5)	**<0.001**
	12 m	94.5 (1.8)	75.2 (1.6)	74.4 (1.3)	**+15.9 (2.9) *p*<0.001**	Follow-up effect	−16.9 (−24.4;−9.4)	**<0.001**	−19.9 (−26.8;−12.9)	**<0.001**
Protein	0 m	1.08 (0.0)	1.07 (0.0)	1.08 (0.0)						
(g/kg/day)	6 m	1.43 (0.0)	1.11 (0.0)	1.13 (0.0)	**+0.35 (0.0) *p*<0.001**	Intervention effect	−0.31 (-.41;-.20)	**<0.001**	−0.31 (-.40;-.21)	**<0.001**
	12 m	1.25 (0.0)	1.08 (0.0)	1.04 (0.0)	**+0.18 (0.0) *p*<0.001**	Follow-up effect	−0.17 (-.28;-.06)	**0.002**	−0.23 (-.33;-.13)	**<0.001**
Protein source										
Protein animal	0 m	49.7 (1.4)	48.2 (1.1)	49.8 (1.0)						
(g/day)	6 m	76.2 (1.4)	49.6 (1.1)	52.4 (1.0)	**+26.6 (2.6) *p*<0.001**	Intervention effect	−25.21 (−31.7;−18.7)	**<0.001**	−23.92 (−30.0;−17.8)	**<0.001**
	12 m	64.4 (1.4)	46.2 ((1.1)	45.6 (1.0)	**+14.7 (2.6) *p*<0.001**	Follow-up effect	−16.76 (−23.5;−10.0)	**<0.001**	−18.97 (−25.2;−12.8)	**<0.001**
Protein plant	0 m	28.8 (0.9)	28.0 (0.7)	28.2 (0.7)						
(g/day)	6 m	30.7 (0.9)	28.6 (0.7)	28.3 (0.7)	+1.89 (1.1) *p*=0.091	Intervention effect	−1.32 (−4.17;1.54)	0.366	−1.66 (−4.34;1.02)	0.224
	12 m	28.1 (0.9)	28.9 (0.7)	28.7 (0.7)	−0.36 (1.1) *p*=0.752	Follow-up effect	+1.25 (−1.73;4.23)	0.411	+0.85 (−1.90;3.60)	0.545
Product groups										
Egg	0 m	2.5 (0.1)	2.5 (0.1)	2.4 (0.1)						
(g/day)	6 m	3.1 (0.1)	2.3 (0.1)	2.0 (0.1)	+0.60 (0.4) *p*=0.157	Intervention effect	−0.83 (−1.89;.23)	0.126	−1.01 (−2.01;.00)	**0.049**
	12 m	4.4 (0.1)	2.5 (0.1)	1.8 (0.1)	**+1.85 (0.4) *p*<0.001**	Follow-up effect	−1.84 (−2.95;-.73)	**0.001**	−2.52 (−3.55;−1.49)	**<0.001**
Fish	0 m	6.0 (0.5)	5.8 (0.3)	6.9 (0.4)						
(g/day)	6 m	9.9 (0.5)	4.4 (0.3)	6.6 (0.4)	**+3.99 (1.3) *p*=0.003**	Intervention effect	−5.32 (−8.63;−2.00)	**0.002**	−4.35 (−7.47;−1.23)	**0.006**
	12 m	9.4 (0.5)	3.6 (0.3)	5.6 (0.4)	**+3.44 (1.4) *p*=0.012**	Follow-up effect	−5.60 (−9.07;−2.13)	**0.002**	−4.72 (−7.93;−1.51)	**0.004**
Meat	0 m	17.7 (0.7)	17.7 (0.8)	16.8 (0.6)						
(g/day)	6 m	25.5 (0.7)	22.2 (0.8)	20.1 (0.6)	**+7.81 (2.1) *p*<0.001**	Intervention effect	−3.27 (−8.45;1.90)	0.215	−4.46 (−9.35;.42)	*0.073*
	12 m	17.6 (0.7)	18.4 (0.8)	14.8 (0.6)	−0.09 (2.1) *p*=0.967	Follow-up effect	+0.75 (−4.66;6.17)	0.785	−1.88 (−6.90;3.13)	0.462
Dairy	0 m	19.7 (1.1)	19.2 (0.7)	20.7 (0.7)						
(g/day)	6 m	33.9 (1.1)	18.1 (0.7)	20.9 (0.7)	**+14.2 (1.4) *p*<0.001**	Intervention effect	−15.33 (−19.0;−11.7)	**<0.001**	−13.95 (−17.4;−10.5)	**<0.001**
	12 m	29.3 (1.1)	19.2 (0.7)	20.1 (0.7)	**+9.65 (1.5) *p*<0.001**	Follow-up effect	−9.71 (−13.5;−5.90)	**<0.001**	−10.24 (−13.8;−6.72)	**<0.001**
Bread	0 m	10.9 (0.5)	10.3 (0.4)	10.3 (0.4)						
(g/day)	6 m	10.6 (0.5)	10.4 (0.4)	10.6 (0.4)	−0.35 (0.6) *p*=0.566	Intervention effect	+0.45 (−1.1;2.00)	0.566	+0.64 (-.82;2.10)	0.390
	12 m	10.6 (0.5)	10.9 (0.4)	10.5 (0.4)	−0.33 (0.6) *p*=0.598	Follow-up effect	+0.93 (-.69;2.54)	0.261	+0.46 (−1.04;1.95)	0.549

Vegetables/fruits	0 m	4.5 (0.2)	4.4 (0.2)	4.4 (0.2)						
(g/day)	6 m	4.6 (0.2)	4.5 (0.2)	4.2 (0.2)	+0.09 (0.4) *p*=0.780	Intervention effect	+0.06 (-.78;.89)	0.891	−0.25 (−1.03;.54)	0.539
	12 m	4.2 (0.2)	4.1 (0.2)	4.4 (0.2)	−0.38 (0.4) *p*=0.268	Follow-up effect	+0.12 (-.75;1.00)	0.768	+0.43 (-.38;1.23)	0.322
Legumes/Soy	0 m	1.1 (0.2)	1.3 (0.2)	1.3 (0.2)						
(g/day)	6 m	1.8 (0.2)	1.5 (0.2)	1.5 (0.2)	+0.73 (0.5) *p*=0.125	Intervention effect	−0.54 (−1.75;.67)	0.381	−0.50 (−1.63;.64)	0.394
	12 m	1.4 (0.2)	1.1 (0.2)	1.7 (0.2)	+0.39 (0.5) *p*=0.429	Follow-up effect	−0.51 (−1.78;.75)	0.428	+0.02 (−1.15;1.19)	0.971
Nuts/seeds	0 m	4.0 (0.2)	3.7 (0.1)	3.9 (0.2)						
(g/day)	6 m	5.8 (0.2)	3.2 (0.1)	3.2 (0.2)	**+1.85 (0.7) *p*=0.011**	Intervention effect	−2.40 (−4.19;-.61)	**0.009**	−2.61 (−4.29;-.92)	**0.002**
	12 m	4.7 (0.2)	3.9 (0.1)	3.6 (0.2)	+0.71 (0.7) *p*=0.344	Follow-up effect	−0.59 (−2.47;1.29)	0.539	−1.04 (−2.77;.69)	0.239
Other	0 m	12.0 (0.6)	11.6 (0.3)	11.6 (0.4)						
(g/day)	6 m	12.4 (0.6)	11.7 (0.3)	12.1 (0.4)	+0.48 (1.0) *p*=0.619	Intervention effect	−0.34 (−2.82;2.13)	0.786	+0.05 (−2.28;2.39)	0.964
	12 m	13.1 (0.6)	11.8 (0.3)	12.1 (0.4)	+1.18 (1.0) *p*=0.240	Follow-up effect	−0.96 (−3.55;1.63)	0.466	−0.63 (−3.03;1.77)	0.609
Dairy detailed										
Cheese	0 m	7.9 (0.3)	7.8 (0.3)	8.1 (0.3)						
(g/day)	6 m	9.9 (0.3)	6.3 (0.3)	8.4 (0.3)	**+1.97 (0.9) *p*=0.034**	Intervention effect	−3.42 (−5.73;−1.12)	**0.004**	−1.65 (−3.82;.51)	0.134
	12 m	9.2 (0.3)	7.6 (0.3)	7.3 (0.3)	+1.30 (1.0) *p*=0.174	Follow-up effect	−1.46 (−3.87;.96)	0.237	−2.06 (−4.28;.17)	*0.070*
Milk	0 m	5.2 (0.6)	5.3 (0.5)	6.0 (0.5)						
(g/day)	6 m	8.4 (0.6)	4.8 (0.5)	6.2 (0.5)	**+3.22 (0.7) *p*<0.001**	Intervention effect	−3.72 (5.47;−1.96)	**<0.001**	−3.05 (−4.69;−1.40)	**<0.001**
	12 m	7.3 (0.6)	5.3 (0.5)	5.9 (0.5)	**+2.14 (0.7) *p*=0.002**	Follow-up effect	−2.09 (−3.92;-.26)	**0.026**	−2.25 (−3.94;-.55)	**0.009**
Quark	0 m	2.5 (0.5)	1.7 (0.3)	2.1 (0.3)						
(g/day)	6 m	10.9 (0.5)	2.0 (0.3)	2.3 (0.3)	**+8.42 (0.8) *p*<0.001**	Intervention effect	−8.17 (−10.22;−6.11)	**<0.001**	−8.17 (−10.10;−6.23)	**<0.001**
	12 m	8.1 (0.5)	1.6 (0.3)	2.3 (0.3)	**+5.55 (0.8) *p*<0.001**	Follow-up effect	−5.67 (−7.82;−3.51)	**<0.001**	−5.31 (−7.30;−3.32)	**<0.001**
Yoghurt	0 m	3.0 (0.3)	4.0 (0.4)	3.6 (0.2)						
(g/day)	6 m	3.3 (0.3)	4.2 (0.4)	2.9 (0.2)	+0.31 (0.5) *p*=0.544	Intervention effect	−0.16 (−1.46;1.13)	0.806	−0.98 (−2.20;.24)	0.115
	12 m	3.7 (0.3)	4.3 (0.4)	3.3 (0.2)	+0.70 (0.5) *p*=0.179	Follow-up effect	−0.46 (−1.81;.89)	0.504	−0.99 (−2.24;.26)	0.120
Dairy other	0 m	0.8 (0.2)	0.7 (0.1)	0.9 (0.1)						
(g/day)	6 m	1.2 (0.2)	0.8 (0.1)	0.9 (0.1)	*+0.43 (0.3) p=0.086*	Intervention effect	−0.33 (-.96;.31)	0.309	−0.43 (−1.02;.17)	0.162
	12 m	0.9 (0.2)	0.4 (0.1)	1.0 (0.1)	+0.13 (0.3) *p*=0.613	Follow-up effect	−0.47 (−1.13;.20)	0.168	+0.01 (-.60;.63)	0.968
Amino acids										
Essential AA	0 m	31.1 (0.7)	30.0 (0.6)	31.1 (0.6)						
(g/day)	6 m	43.1 (0.7)	30.6 (0.6)	31.9 (0.6)	**+11.97 (1.2) *p*<0.001**	Intervention effect	−11.38 (−14.47;−8.30)	**<0.001**	−11.18 (−14.07;−8.28)	**<0.001**
	12 m	37.0 (0.7)	30.3 (0.6)	29.8 (0.6)	**+5.81 (1.2) *p*<0.001**	Follow-up effect	−5.53 (−8.76;−2.31)	**0.001**	−7.05 (−10.03;−4.08)	**<0.001**
Non-essential AA[b]	0 m	35.0 (0.8)	33.6 (0.7)	34.7 (0.6)						
(g/day)	6 m	46.8 (0.8)	34.0 (0.7)	35.8 (0.6)	**+11.84 (1.3) *p*<0.001**	Intervention effect	−11.46 (−14.78;−8.14)	**<0.001**	−10.80 (−13.92;−7.68)	**<0.001**
	12 m	40.1 (0.8)	34.6 (0.7)	33.6 (0.6)	**+5.10 (1.4) *p*<0.001**	Follow-up effect	−4.06 (−7.53;-.58)	**0.022**	−6.17 (−9.38;−2.97)	**0.001**
BCAA	0 m	14.2 (0.3)	13.7 (0.3)	14.3 (0.3)						
(g/day)	6 m	19.7 (0.3)	13.9 (0.3)	14.6 (0.3)	**+5.44 (0.5) *p*<0.001**	Intervention effect	−5.25 (−6.64;−3.86)	**<0.001**	−5.15 (−6.46;−3.84)	**<0.001**
	12 m	17.0 (0.3)	14.0 (0.3)	13.7 (0.3)	**+2.72 (0.6) *p*<0.001**	Follow-up effect	−2.45 (−3.91;−0.99)	**0.001**	−3.24 (−4.58;−1.90)	**<0.001**
Leucine	0 m	6.3 (0.1)	6.0 (0.1)	6.3 (0.1)						
(g/day)	6 m	8.7 (0.1)	6.1 (0.1)	6.4 (0.1)	**+2.39 (0.2) *p*<0.001**	Intervention effect	−2.30 (−2.92;−1.67)	**<0.001**	−2.27 (−2.84;−1.68)	**<0.001**
	12 m	7.4 (0.1)	6.2 (0.1)	6.1 (0.1)	**+1.17 (0.2) *p*<0.001**	Follow-up effect	−1.03 (−1.68;-.39)	**0.002**	−1.40 (−1.99;-.80)	**<0.001**
Protein at eating occasions										
MM 1 - breakfast	0 m	13.1 (0.6)	13.2 (0.6)	12.9 (0.5)						
	6 m	19.2 (0.6)	13.3 (0.6)	13.0 (0.5)	**+6.15 (1.0) *p*<0.001**	Intervention effect	−6.04 (−8.51;−3.58)	**<0.001**	−6.07 (−8.39;−3.75)	**<0.001**
	12 m	19.8 (0.6)	13.3 (0.6)	12.5 (0.5)	**+6.73 (1.1) *p*<0.001**	Follow-up effect	−6.66 (−9.24;−4.08)	**<0.001**	−7.16 (−9.54;−4.78)	**<0.001**
MM 2 - morning	0 m	3.9 (0.3)	3.4 (0.2)	3.7 (0.2)						

	6 m	5.6 (0.3)	3.2 (0.2)	3.9 (0.2)	**+1.77 (0.7) *p*=0.007**	Intervention effect	−1.96 (−3.6;-.33)	**0.019**	−1.56 (−3.10;-.02)	**0.047**
	12 m	5.6 (0.3)	3.5 (0.2)	3.3 (0.2)	**+1.69 (0.7) *p*=0.012**	Follow-up effect	−1.54 (−3.26;.17)	*0.078*	−2.16 (−3.74;-.58)	**0.007**
MM 3 - lunch	0 m	18.3 (0.7)	18.0 (0.5)	18.8 (0.5)						
	6 m	25.8 (0.7)	17.4 (0.5)	19.3 (0.5)	**+7.48 (1.2) *p*<0.001**	Intervention effect	−8.06 (−11.12;−5.00)	**<0.001**	−6.97 (−9.85;−4.10)	**<0.001**
	12 m	22.5 (0.7)	19.5 (0.5)	18.6 (0.5)	**+4.21 (1.2) *p*=0.001**	Follow-up effect	−2.73 (−5.93;.47)	0.095	−4.35 (−7.31;−1.40)	**0.004**
MM 4 - afternoon	0 m	6.5 (0.3)	6.1 (0.3)	6.1 (0.2)						
	6 m	9.0 (0.3)	5.9 (0.3)	6.2 (0.2)	**+2.54 (1.0) *p*=0.009**	Intervention effect	−2.68 (−5.07;-.29)	**0.028**	−2.41 (−4.66;-.16)	**0.036**
	12 m	6.9 (0.3)	4.1 (0.3)	5.2 (0.2)	+0.47 (1.0) *p*=0.639	Follow-up effect	−2.40 (−4.91;.11)	*0.061*	−1.33 (−3.65;1.0)	0.260
MM 5 - diner	0 m	34.1 (0.7)	33.7 (0.7)	34.6 (0.7)						
	6 m	44.7 (0.7)	36.6 (0.7)	37.3 (0.7)	**+10.61 (1.9) *p*<0.001**	Intervention effect	−7.67 (−12.54;−2.97)	**0.002**	−7.89 (−12.49;−3.28)	**0.001**
	12 m	35.4 (0.7)	32.2 (0.7)	32.1 (0.7)	+1.25 (2.0) *p*=0.529	Follow-up effect	−2.68 (−7.79;2.42)	0.303	−3.78 (−8.51;.95)	0.117
MM 6 - evening	0 m	5.1 (0.4)	4.5 (0.3)	4.7 (0.2)						
	6 m	7.2 (0.4)	3.8 (0.3)	4.3 (0.2)	**+2.10 (0.7) *p*=0.004**	Intervention effect	−2.79 (−4.63;-.96)	**0.003**	−2.52 (−4.25;-.79)	**0.004**
	12 m	7.3 (0.4)	4.3 (0.3)	4.7 (0.2)	**+2.25 (0.7) *p*=0.003**	Follow-up effect	−2.40 (−4.31;-.48)	**0.014**	−2.20 (−3.97;-.42)	**0.015**

* P values for the comparison among the groups from baseline to 6 months and 12 months were calculated with the use of mixed-model analysis of repeated measures. Fixed factors include time and group*time interaction. Random intercepts include cluster and subject. Unless otherwise noted, no covariates added. [a] Covariates Sex and Age were added. [b] Model without cluster. CON, Control group (*n*=84); HBex, Home-based exercise training group (*n*=63); HBex-Pro, Home-based exercise training with dietary protein counselling group (*n*=65).
# HBex-Pro is the reference group. † Difference in mean scores HBex-Pro vs HBex. ‡ Difference in mean scores HBex-Pro vs CON.

Supplementary Table S2. Frequencies of leucine compliers per meal (≥2.5 g) for all groups

Article title: Digitally supported dietary protein counselling changes dietary protein intake, sources and distribution in community-dwelling older adults

Journal name: Nutrients

Author names: Jantine van den Helder, Sjors Verlaan, Michael Tieland, Jorinde Scholten, Sumit Mehra, Bart Visser, Ben J.A. Kröse, Raoul H.H. Engelbert and Peter J.M. Weijs.

Corresponding Author: Jantine van den Helder, Faculty of Sports and Nutrition, Center of Expertise Urban Vitality, Amsterdam University of Applied Sciences, Dokter Meurerlaan 8, 1067 SM, Amsterdam, the Netherlands

T +31621155919 | Email j.e.m.van.den.helder@hva.nl

Table S2. Frequencies of leucine compliers per meal (≥2.5 g) for all groups.

	HBex-Pro			HBex			CON		
	0 m (*n*=65)	6 m (*n*=45)	12 m (*n*=42)	0 m (*n*=63)	6 m (*n*=55)	12 m (*n*=48)	0 m (*n*=84)	6 m (*n*=81)	12 m (*n*=77)
0 meals	25 (39)	4 (9)	14 (33)	36 (57)	21 (38)	21 (44)	33 (39)	33 (41)	33 (43)
1 meal	34 (52)	25 (56)	20 (48)	23 (37)	29 (53)	23 (48)	43 (51)	41 (51)	39 (51)
2 meals	6 (9)	13 (29)	7 (17)	4 (6)	5 (9)	3 (6)	8 (10)	7 (9)	5 (6)
3 meals		3 (7)	1 (2)			1 (2)			

No. and (%) of compliers to the leucine per meal target (≥2.5 gram). CON, Control group; HBex, Home-based exercise training group; HBex-Pro, Home-based exercise training with dietary protein counselling group.

Supplementary Table S3. Frequencies of protein compliers per meal (≥25 g) for all groups

Article title: Digitally supported dietary protein counselling changes dietary protein intake, sources and distribution in community-dwelling older adults

Journal name: Nutrients

Author names: Jantine van den Helder, Sjors Verlaan, Michael Tieland, Jorinde Scholten, Sumit Mehra, Bart Visser, Ben J.A. Kröse, Raoul H.H. Engelbert and Peter J.M. Weijs.

Corresponding Author: Jantine van den Helder, Faculty of Sports and Nutrition, Center of Expertise Urban Vitality, Amsterdam University of Applied Sciences, Dokter Meurerlaan 8, 1067 SM, Amsterdam, the Netherlands

T +31621155919 | Email j.e.m.van.den.helder@hva.nl

Table S3. Frequencies of compliers of protein per meal (≥25 g) for all groups.

	HBex-Pro			HBex			CON		
	0 m (*n*=65)	6 m (*n*=45)	12 m (*n*=42)	0 m (*n*=63)	6 m (*n*=55)	12 m (*n*=48)	0 m (*n*=84)	6 m (*n*=81)	12 m (*n*=77)
0 meals	10 (15)	5 (11)	10 (24)	14 (22)	10 (18)	10 (21)	16 (19)	10 (12)	18 (23)
1 meal	39 (60)	16 (36)	19 (45)	37 (59)	35 (64)	26 (54)	46 (55)	54 (67)	38 (49)
2 meals	14 (22)	15 (33)	7 (17)	11 (17)	9 (16)	12 (25)	21 (25)	16 (20)	21 (27)
≥ 3 meals	2 (3)	9 (20)	6 (14)	1 (2)	1 (2)	0 (0)	1 (1)	1 (1)	0 (0)

No. and (%) of compliers to the protein per meal target (≥25 gram). CON, Control group; HBex, Home-based exercise training group; HBex-Pro, Home-based exercise training with dietary protein counselling group.
